# Autonomic dysfunction after moderate-to-severe traumatic brain injury: symptom spectrum and clinical testing outcomes

**DOI:** 10.1136/bmjno-2022-000308

**Published:** 2022-04-24

**Authors:** Lucia M Li, Ekawat Vichayanrat, Martina del Giovane, Helen Hoi Lun Lai, Valeria Iodice

**Affiliations:** 1 Division of Brain Sciences, Imperial College, London, UK; 2 UK Dementia Research Institute Care Research and Technology Centre, Imperial College London and the University of Surrey, London, UK; 3 Autonomics Unit, National Hospital for Neurology and Neurosurgery, London, UK

**Keywords:** autonomic function | traumatic brain injury | sympathetic | parasympathetic | health outcome

## Abstract

**Background:**

Survivors of moderate-to-severe traumatic brain injury (msTBI) frequently experience troublesome unexplained somatic symptoms. Autonomic dysfunction may contribute to these symptoms. However, there is no previous study of clinical subjective and objective autonomic dysfunction in msTBI.

**Methods:**

We present results from two groups of patients with msTBI. The first, a case–control comparative study, comprises prospectively recruited msTBI outpatients, in whom we measured burden of autonomic symptoms using the Composite Autonomic Symptom Score (COMPASS31) questionnaire. The second, a descriptive case series, comprises retrospectively identified msTBI outpatients who had formal clinical autonomic function testing at a national referral autonomics unit.

**Results:**

Group 1 comprises 39 patients with msTBI (10F:20M, median age 40 years, range 19–76), median time from injury 19 months (range 6–299) and 44 controls (22F:22M, median age 45, range 25–71). Patients had significantly higher mean weighted total COMPASS-31 score than controls (p<0.001), and higher gastrointestinal, orthostatic and secretomotor subscores (corrected p<0.05). Total COMPASS31 score inversely correlated with subjective rating of general health (p<0.001, r_s_=−0.84). Group 2 comprises 18 patients with msTBI (7F:11M, median age 44 years, range 21–64), median time from injury 57.5 months (range 2–416). Clinical autonomic function testing revealed a broad spectrum of autonomic dysfunction in 13/18 patients.

**Conclusions:**

There is clinically relevant autonomic dysfunction after msTBI, even at the chronic stage. We advocate for routine enquiry about potential autonomic symptoms, and demonstrate the utility of formal autonomic testing in providing diagnoses. Larger prospective studies are warranted, which should explore the causes and clinical correlates of post-TBI autonomic dysfunction.

**Key messages**

**What is already known on this topic**

- Patients with severe traumatic brain injury (TBI) can experience sympathetic hyperactivity, a form of autonomic dysfunction, in the acute stages. In the chronic stage of injury, patientswith TBI have many hitherto unexplained physical symptoms.

**What this study adds**

- We find evidence for a broad range of autonomic symptoms, and objective evidence of both parasympathetic and sympathetic dysfunction in moderate-to-severe patients with TBI. This dysfunction exists even in patients who are in the chronic stage of injury, ambulant and functionally independent.

**How this study might affect research, practice and/or policy**

- We advocate for routine enquiry about autonomic symptoms in survivors of moderate-to-severe TBI. Further studies should map the epidemiology, and investigate the causes of postinjury autonomic dysfunction.

## Introduction

Moderate-to-severe traumatic brain injury (msTBI) is known to cause high rates of chronic neurologic, cognitive and behavioural sequelae. What is less appreciated is that TBI survivors often also report long-lasting systemic symptoms, many of which are suggestive of autonomic nervous system (ANS) dysfunction.^1^ Indeed, changes in heart rate (HR) variability, an experimental measure of cardiovascular ANS function, has been found in a number of studies of concussion and mild TBI, with one study reporting a positive correlation between TBI severity and degree of both sympathetic and parasympathetic impairment.^2–5^

Orthostatic symptoms, presyncopal and syncopal episodes are those most likely to be recognised as being autonomically-mediated. However, ANS dysfunction can manifest in myriad additional symptoms that can negatively impact daily function and quality of life, such as abnormal sweating, nausea and bloating, constipation and diarrhoea, dry eyes and mouth, changes in skin colour and thermodysregulation, erectile dysfunction, visual blurring and photosensitivity.^1^ The Composite Autonomic Symptom Score-31 (COMPASS-31) tool is a simple-to-administer questionnaire which quantitatively assesses the symptomatic burden of autonomic dysfunction.^6^ Clinical autonomic function tests non-invasively assess the integrity of the ANS across multiple modalities, including cardiovagal, sudomotor and adrenergic function, and have shown utility in characterising and tracking ANS dysfunction^7^

Autonomic dysfunction in the acute phase of severe TBI is well described in the syndrome of Paroxysmal Sympathetic Hyperactivity.^8^ However, beyond one or two case studies, no study has investigated clinically relevant autonomic dysfunction in the postacute period after TBI. We present the results of a clinical investigation in two groups of patients with msTBI. First, we collected quantitative symptom assessments in a cohort prospectively identified from a national TBI clinic. Second, we present clinical autonomic function testing results from a case series of patients retrospectively identified from a national referral Autonomic centre. We hypothesise that patients in the post-acute period of msTBI (1) experience a high burden of clinical autonomic symptoms, which (2) is associated with other health measures and (3) have objectively measurable sympathetic and parasympathetic dysfunction.

## Methods

### Study design and participants

**Figure 1:**
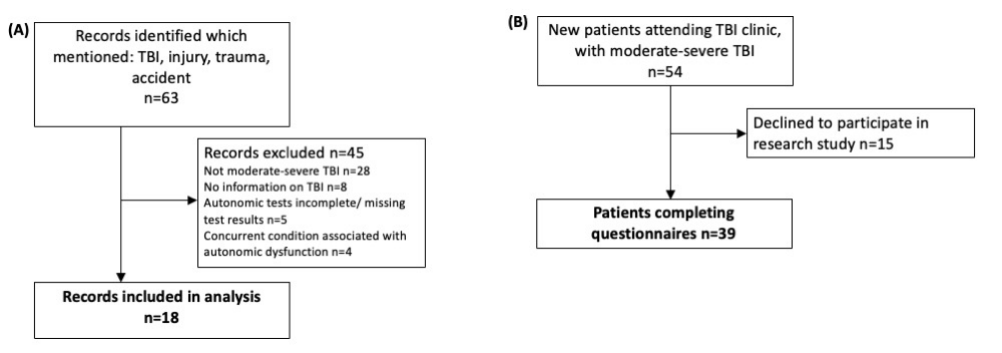
Flow chart denoting (A) records included in retrospective analysis of autonomic testing, and (B) patients included in prospective symptom assessment. TBI, traumatic brain injury.

We investigated two groups of patients with msTBI, defined on Mayo Classification,^9^ in a cross-sectional study. The first, a retrospective case series, was identified from a database of all patients undergoing autonomic tests at a tertiary autonomics clinic between 2010 and 2020. LML, EV and VI had full access to this database. Test reports were screened with the following terms: TBI, injury, bleed, haemorrhage, haemorrhage, trauma* (wild card to bring up, eg, ‘trauma’, ‘traumatic’), accident. We verified injury and medical history details by reviewing the individual’s electronic health records. Inclusion criteria were: history consistent with msTBI and injury preceded the symptoms for referral. Exclusion criteria were: TBI sustained in the context of orthostatic symptoms, concurrent spinal injury, inadequate information to determine TBI severity or timeline relative to symptoms, antecedent or concurrent vestibular diagnosis (eg, vestibular migraine, benign paroxysmal postural vertigo) where dizziness was the only symptom, and antecedent or concurrent diagnoses which could explain symptoms (eg, long-standing diabetes, Parkinson’s disease) (figure 1A).

The second, prospective cohort was recruited from an adult TBI clinic in January–August 2021. To mitigate selection bias, all new patients with ms^9^-TBI were invited to complete the COMPASS-31 questionnaire, irrespective of whether they reported symptoms suspicious for ANS dysfunction, and answer questions about their perception of their own health. A cohort of 44 control participants also completed the questionnaire, recruited by email over 2 weeks from a volunteer database. Exclusion criteria were: acquired brain injury of any aetiology, significant current medical diagnosis. In order to partially account for medications and TBI-related comorbidity, control participants were not excluded for being on: analgesic medications, anxiolytics and antidepressants or birth control.

### Autonomic function testing: acquisition and analysis

Before testing, patients are asked to refrain from: heavy exercise for 24 hours, alcohol and caffeine intake for 12 hours, and eating for 1 hour. All patients were self-ambulant, without prolonged daily recumbency and attended as outpatients. The clinical testing protocol included:

- Head up tilt—from supine to 60° head up, continued for 10 min or until patient could no longer tolerate or symptoms of imminent syncope. Beat-to-beat HR and blood pressure (BP) were recorded with a Finapress or Finometer. If clinical history suggested situational syncope, venepuncture was performed as an additional provocation during the tilt.
- Supine/tilted norepinephrine (NA) levels—plasma levels used as a biochemical marker of sympathetic neural activity, measured using high-performance liquid chromatography.
- Respiratory sinus arrythmia (HR_DB_)—parasympathetic function was assessed by HR response to deep breathing. Patients breathed at six breaths/minute for 1 min, and were coached on how long each breath should take. HR_DB_ was the average of the differences between the minimum and maximum HRs over the six breaths, and classified as abnormal if outside of the age-appropriate range.^10^
- Valsalva manoeuvre—participants performed a forced expiration, using a 2 mL syringe tube, for 15 s against a fixed expiratory pressure of 40 mm Hg for 10–15 s. The BP response, indicative of adrenergic function, was classified as normal/abnormal by the performing clinical scientist, and confirmed by VI and EV. The Valsalva ratio (VR), reflecting both parasympathetic and sympathetic function, is the ratio of the maximum HR that develops in response to manoeuvre-induced BP reduction (following phases II/III), divided by the minimum HR that results from the manoeuvre-induced BP overshoot (occurring within 30 s of phase IV peak).^11^ The VR was classified as abnormal if it was outside of the age-appropriate range.^10^

Individual patient results are described.

### Prospective questionnaire symptom assessment

The COMPASS-31 questionnaire was used to assess presence and severity of autonomic symptoms,^6^ and assesses several domains: orthostatic, vasomotor (sweating/skin changes), secretomotor (dry eyes or mouth), gastrointestinal (postprandial symptoms, constipation, diarrhoea), bladder (difficulty passing urine, incontinence) and pupillomotor (photosensitivity, blurred vision). Each domain is scored separately, multiplied by a weighting factor and then combined (maximum score=100). This questionnaire has been previously validated and used to quantitatively assess burden of autonomic symptoms in autonomic disorders, including those with intermittent symptoms,^12 13^ with good sensitivity and discrimination value for autonomically mediated symptoms.^14–16^ Patients completed the COMPASS questionnaire via phone (LML, MdG or HHLL) or online. A small number were completed face-to-face (MdG or HHLL). Healthy controls completed the questionnaire online.

All patients were invited to answer the following additional questions:

- Subjective rating of general health state: “Overall how would you rate your health?” (Visual Analogue Scale: 0=poor – 100=excellent)
- Pain: ‘I have physical pain or discomfort’. (5-category Likert Scale: 0=‘not true’ – 4=’definitely true’)
- Fatigue: ‘Are you tired?’ (Visual Analogue Scale: 0=very tired – 100=no tiredness at all)
- Depression: ‘Do you feel sad or depressed?’ (Visual Analogue Scale: 0=very sad or depressed – 100=not depressed or sad at all)
- Anxiety: ‘Do you feel anxious?’ (Visual analogue scale: 0=very anxious – 100=not anxious at all)
- Health outlook: ‘Do you think things are getting better?’ (Visual analogue scale: 0=things are getting worse – 100=things are getting better)

Visual analogue responses were captured by participants dragging a cursor along a line.

We compared COMPASS-31 scores between patients with TBI and controls using unpaired Student’s t-test, and tested for correlations between COMPASS-31 score and other scores using Spearman correlations. Bonferroni correction was used to correct for number of t-tests or correlations performed. All statistical tests were performed using RStudio (RStudio PBC, V.2021.09.0).

### Data availability

Raw scores from the COMPASS31 questionnaire and anonymised clinical autonomic function testing reports from the retrospective cohort are available on request to the corresponding author.

## Results

### Prospective symptom burden assessment cohort

**Figure 2:**
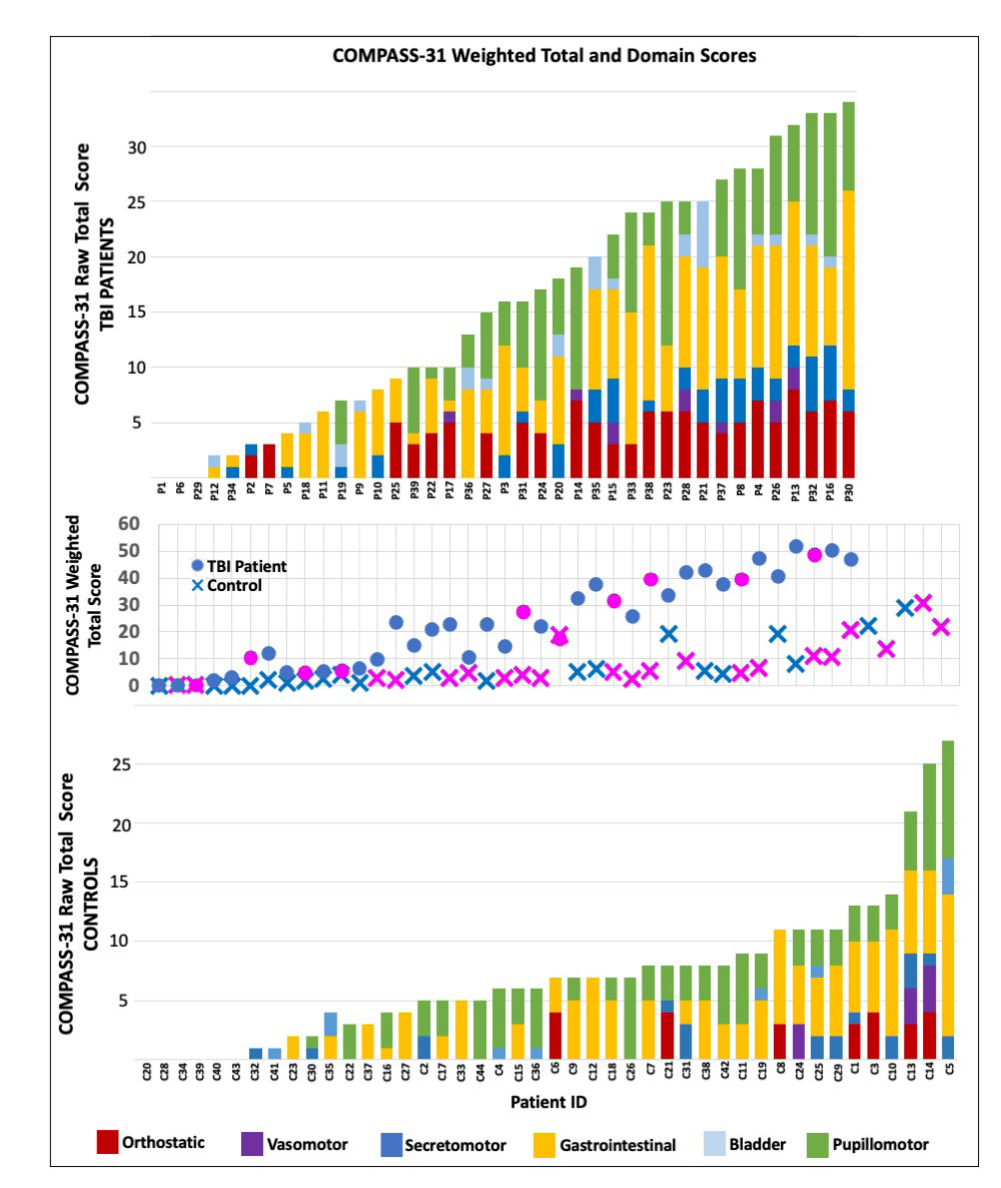
Symptom burden assessed with COMPASS-31, with weighted total score for each patient (middle panel: circles are controls and crosses are patients with TBI, with pink for female and blue for male) and corresponding domain subscore distribution (top panel for patients with TBI, bottom panel for controls), ordered by RAW total score. COMPASS-31, Composite Autonomic Symptom Score; TBI, traumatic brain injury.

Thirty-nine patients with TBI (10F/29M, median age 40 years, range 19–76) (figure 1B) and 44 controls (22F/22M, median age 45 years, range 25–71) completed the questionnaire (online supplemental table 1A, B). The median time since injury was 19 months, (range 6–299). Patients with TBI reported multidomain symptom burden (figure 2). Only 3 patients did not report any symptoms, with 22 of 39 patients having a weighted total score >20, and 21 of 39 patients reporting symptoms in at least 3 subdomains. Symptoms in the gastrointestinal subdomain was most commonly reported (32/39 patients), followed by orthostatic symptoms (25/39 patients), then pupillomotor (24/39 patients), then secretomotor (21/39 patients). Bladder (15/39) and vasomotor symptoms were least common (7/39 patients).

10.1136/bmjno-2022-000308.supp1 Supplementary data

The TBI cohort had significantly higher mean weighted COMPASS31 scores than healthy controls (TBI 23.3 (SD 16.65) vs conrols 7.33 (SD 8.10), p<*0.001*). Patients with TBI also had significantly higher mean symptom subscores in orthostatic (TBI 3.18 (SD 2.69) vs controls 0.57 (SD 1.33)), secretomotor (TBI 1.33 (SD 1.58) vs controls 0.48 (SD 0.88)) and gastrointestinal symptoms (TBI 6.03 (4.77) vs controls 3.09 (SD 3.05)) (corrected p<0.*05*). Patients with TBI also had higher average raw symptom subscores in bladder (TBI 0.67 (SD 1.18) vs control 0.23 (SD 0.60)) and pupillomotor (TBI 4.18 (SD 4.29) vs control 2.5 (SD 2.51)) symptoms, but this was not significant after correction for multiple comparisons. There was no significant difference between TBI and healthy controls in the mean vasomotor sub-score (TBI 0.28 (SD 0.65) vs control 0.23 (SD 0.86)).

**Figure 3:**
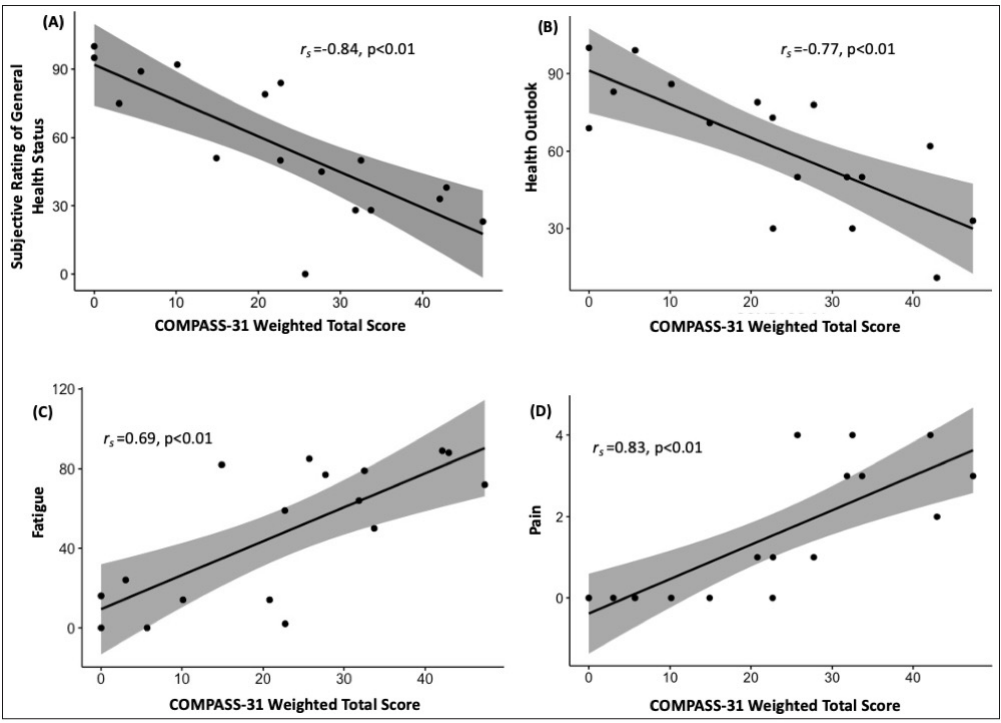
Relationship between the COMPASS-31 weighted total score and (A) subjective rating of general health status, (B) health outlook, (C) fatigue and (D) pain. COMPASS-31, Composite Autonomic Symptom Score.

Seventeen patients also completed additional questions. There were very strong negative correlations between weighted total COMPASS31 score and Subjective Rating of General Health state (r_s_=−0.84, p<0.01), and Health Outlook (r_s_=−0.77, p<0.01). Conversely, there were very strong positive correlations between weighted total COMPASS31 score and Fatigue (r_s_=0.69, p<0.01) and Pain (r_s_=0.83, p<0.01) ratings (figure 3). There were no significant correlations between COMPASS31 score with anxiety or depression ratings, age or time since injury.

### Retrospective autonomic function testing cohort

**Figure 4:**
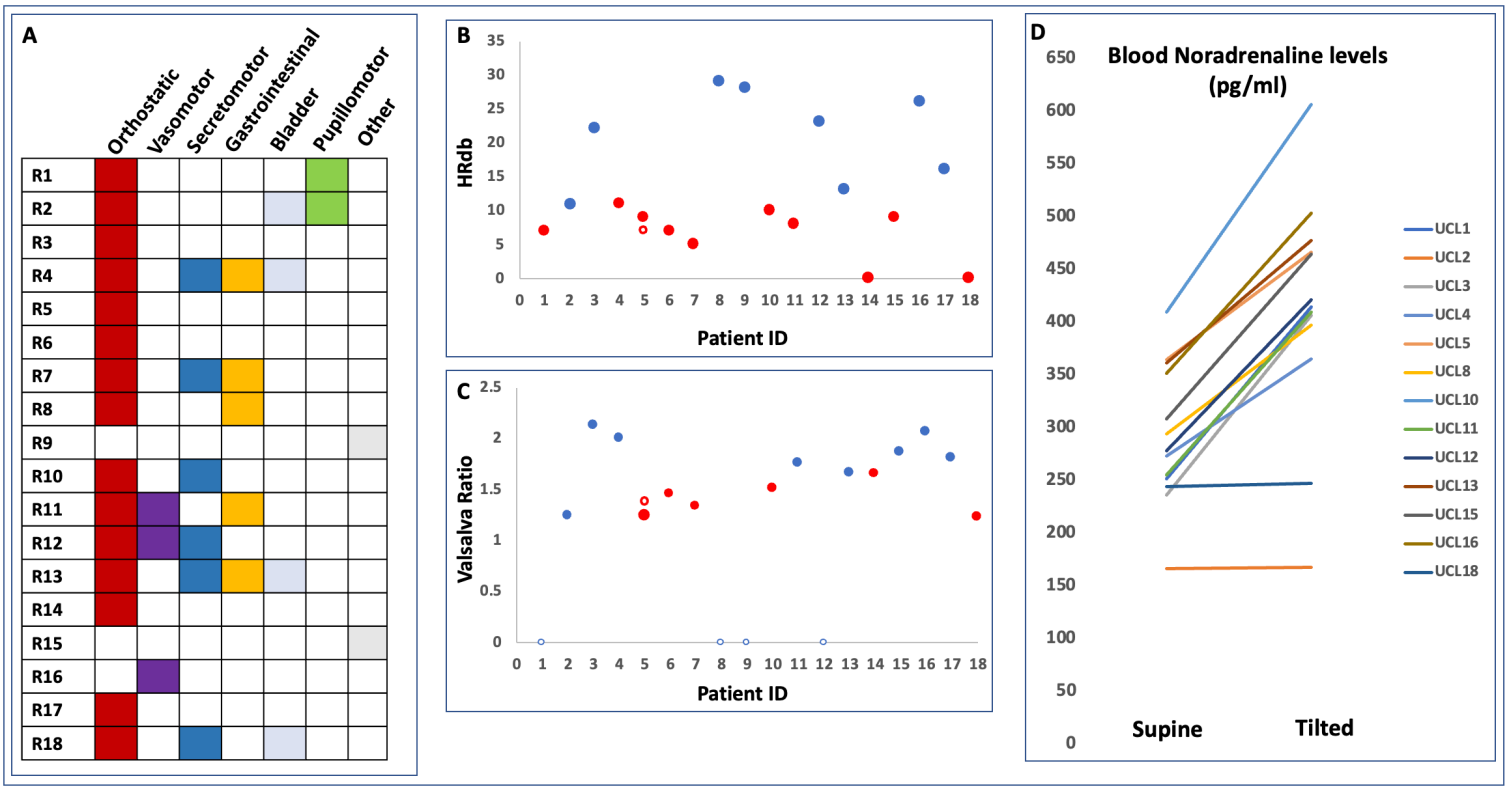
(A) Symptoms reported by each patient, categorised into symptom type. (B) HR_DB_ for each patient. Where there was no respiratory sinus arrhythmia noted, the value of HR_DB_ is noted as 0. A red dot denotes an abnormal age-corrected value. The unfilled red circle (R5) denotes the value from follow-up testing. (C) Valsalva ratio for each patient. A red dot denotes an abnormal age-corrected value. The unfilled red circle (R5) denotes the value from follow-up testing. An open blue circle denotes Valsava manoeuvre not completed. (D) Supine and tilted blood norepinephrine levels for each patient, where available. HR, heart rate.

A total of 18 patients’ records were identified for further analysis (7F/11M, median age at testing 44 years, range 21–64) (figure 1, online supplemental table 1C). All but one patient were in the chronic phase of injury (median time between TBI and first testing 65 months, range 20–416); patient R14 was tested at 2 months after injury, but after discharge from acute care. The median time between onset of symptoms to first referral for autonomic investigations was 44 months (range: 14–194 months). Most patients (12/18) reported that their symptoms had started since the injury. In this cohort, patients were most likely to have been referred for orthostatic symptoms such as orthostatic intolerance or collapse (15/18 patients) (figure 4A). Sweating changes was also a prominent reason for referral (6/18 patients). Two patients had been referred as part of work-up for a specific symptom, for which autonomic dysfunction was considered in the differential: erectile dysfunction (R9) and non-specific fatigue and malaise (R15).

Autonomic Function Testing in this cohort demonstrated a broad spectrum of dysfunction. Abnormal parasympathetic function, as denoted by absent or low respiratory sinus arrhythmia (measured with HR_DB_), was present in 10 of 18 patients (figure 4B). Mixed dysfunction, as denoted by low VR, was present in 6 of 18 patients, all of which were included in those with abnormal HR_DB_ (figure 4C). An appropriate rise in blood NA levels on tilt was absent in 2 patients, suggestive of sympathetic dysfunction (R2, R18) (figure 4D), one of whom (R18) also had abnormal HR_DB_ and VR. An abnormal BP profile on the Valsalva manoeuvre, indicative of adrenergic dysfunction, was noted in R2.

**Figure 5:**
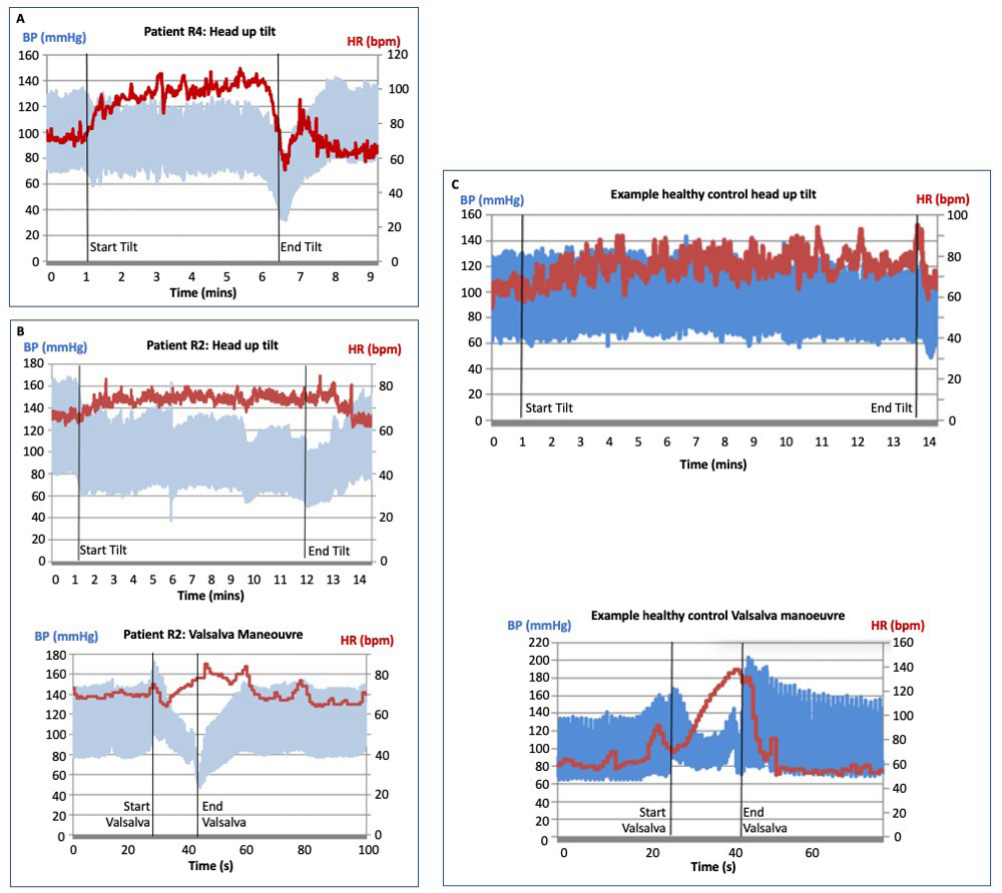
(A) Abnormal cardiovascular responses during head up tilt for patient R4, showing a significant rise in HR on tilt consistent with postural orthostatic tachycardiac syndrome, followed by a drop in both HR and BP supporting a diagnosis of vasovagal syncope. (B) Abnormal cardiovascular responses during the head up tilt (top panel) and Valsalva Manoeuvre (bottom panel) for patient R2 (C) example traces from a healthy control participant showing appropriate HR and bp responses during the head up tilt (top panel) and during the Valsalva manoeuvre (bottom panel). BP, blood pressure; HR, heart rate.

After head-up tilt testing, five patients received confirmed diagnoses of vasovagal syncope (R3, R4, R5, R8, R11), and experienced symptomatic drops in BP on tilt table testing, consistent with reported clinical histories of syncope/pre-syncope starting after their msTBI. Two of these were spontaneous while three were provoked by venepuncture or blood removal from an in situ cannula. R4 also received a diagnosis of postural orthostatic tachycardiac syndrome (POTS), due to a sustained HR rise of >30 on supine/standing measures, and an HR rise of 32 at 4 min of tilt associated with symptoms of orthostatic intolerance (figure 5A).

One patient (R2) received a diagnosis of neurogenic orthostatic hypotension. He experienced a symptomatic BP drop with mild reflex HR increase during head-up tilt (figure 5B, top), suggestive of sympathetic vasoconstrictor failure with sparing of parasympathetic cardiac vagal function (normal HR variability to deep breathing). He also had abnormal BP response during the Valsalva manoeuvre (figure 5B, bottom).

Five additional patients experienced symptoms during head-up tilt without objective significant change in BP or HR. R1, R7, R9, R12 reported ‘dizziness’ while tilted, and R6 had a psychogenic non-syncopal collapse while tilted.

Five patients did not have any abnormalities on routine autonomic function testing (R9, R12, R13, R16, R17). Patient R16 was discovered to have an alternative diagnosis, when nerve conduction studies showed evidence of central somatosensory pathway damage in the limb experiencing vasomotor symptoms.

### Illustrative case: R5

Patient R5 (male in his 40 s) sustained a msTBI in his early 30s. As well as memory and attention problems, he also reported multiple ‘collapses’ with ‘loss of consciousness’, usually triggered by postural change, which had been occurring since his injury. Review and investigation by an epileptologist had concluded that his collapses were not epileptic. He had normal echocardiogram and 24 hours Holter, and was being considered for a Reveal device. He had no other medical history, and not on medication. He had autonomic function tests, including a prolonged tilt. He had a normal BP and HR profile on tilt table assessment without evidence of orthostatic hypotension or autonomic intermittent disorder. There was evidence for mild parasympathetic impairment as documented by both his VR and HR_DB_ below the range for his age (VR=1.26, HR_DB_=9).^10^ His symptoms persisted and he underwent follow-up autonomic function tests 2 years later. Testing confirmed the evidence of further worsening of HR variability to deep breathing (HR_DB_=7), and he also experienced a symptomatic drop in BP after 8 min of head up tilt. In view of his clinical history consistent with syncope and the test results, the patient was treated in our syncope clinic.

## Discussion

We provide evidence for clinically significant autonomic dysfunction after msTBI in two separate outpatient cohorts, with sex and age constitution typical of TBI. The prospective cohort showed multidomain autonomic symptom burden, which correlated with fatigue, pain and negative perception of health status and outlook. Furthermore, there was objective autonomic dysfunction, both sympathetic and parasympathetic, in a retrospectively identified TBI cohort.

While the presence and impact of cognitive and psychiatric symptoms is increasingly recognised in patients with chronic msTBI, there is less recognition of the systemic effects of TBI. Although somatic symptoms are widely reported,^17^ little is known about their causes or consequences. Studies assessing HR variability in small groups of patients with TBI suggests that cardiovascular autonomic dysfunction persists in chronic TBI, and may contribute to clinically relevant symptoms.^3 11 18^ Our retrospective cohort presented with mild autonomic cardiovascular dysfunction within the spectrum of intermittent autonomic dysfunction, and specifically, either new onset vasovagal syncope or postural tachycardia syndrome. Our prospective cohort reported a high burden of symptoms that could be explained by autonomic dysfunction, which also correlated with other symptom and health measures. Our study extends the current literature by demonstrating that objective and clinically relevant autonomic dysfunction is observed after msTBI. Furthermore, we show that this exists in fully ambulant patients with msTBI many months or years after their initial injury, and whom many might assume to be ‘fully recovered’.

Our findings have important clinical implications. Having a high burden of autonomic symptoms impacts on other symptoms and subjective view of health. We also demonstrate that formal autonomic testing, repeated if necessary, can help provide diagnostic clarity in this cohort, especially if there are persistent unexplained symptoms. This enables both appropriate management of the patient’s symptoms and the avoidance of further diagnostic burden. Intermittent autonomic dysfunction, such as vasovagal syncope, is treatable. Timely identification and management are likely to improve long-term quality of life in these patients. Our results suggest that a COMPASS-31 of >30 might be most discriminatory between TBI and non-TB, but further work is required to understand how best to identify patients in whom autonomic function testing would have the highest yield.

It is interesting to note that the retrospective cohort were largely referred for orthostatic symptoms, whereas the symptoms reported by patients in the prospective cohort included a high burden of other types of symptoms, notably gastrointestinal (ie, bloating and nausea after meals, constipation and diarrhoea) symptoms. This may be because postural symptoms are more readily recognised by non-specialists as being potentially mediated by autonomic dysfunction. It is possible that symptoms captured by the COMPASS31 questionnaire due to other, non-autonomic or non-TBI causes, given that some symptoms were also reported by non-TBI controls. However, orthostatic, gastrointestinal and secretomotor scores are significantly higher in TBI than controls, suggesting that this cannot be wholly the case. Indeed, it is just as possible that some post-TBI symptoms are considered non-specific or even misattributed. For example, the high burden of gastrointestinal symptoms may reflect damage to central autonomic pathways, such as the motor nuclei of the vagus nerve, due to brainstem white matter traumatic damage, a mechanism that may be underappreciated by non-autonomic specialists. Indeed, the majority of patients referred for autonomic testing reported experiencing symptoms ‘ever since their injury’, but were not referred until years later. Therefore, it is highly likely that post-TBI autonomic symptoms are under-recognised, and opportunities for further investigation and management are missed.

Autonomic dysfunction after TBI may occur through various mechanisms. The hallmark of msTBI, white matter injury, is caused by axonal shearing due to injury forces and continues due to inflammation and delayed axonal degeneration in the chronic period, and results in network disruption.^19 20^ Autonomic dysfunction may occur due to injury to regions of the central autonomic network (CAN^1^) or their connecting white matter tracts. Brainstem nuclei and white matter connections to and from thalamic and basal ganglia regions may be particularly vulnerable to damage, and underlies catecholaminergic dysfunction contributing to cognitive dysfunction post-TBI.^21 22^ Given the importance of brainstem, thalamic and basal ganglia circuits to autonomic function, injury to these white matter tracts may cause centrally mediated autonomic dysfunction after TBI. TBI may also lead to autonomic dysfunction through indirect mechanisms, for example, interacting with an underlying vulnerability, such as tendency towards hypermobility, or by increasing the risk of conditions independently associated with autonomic dysfunction, such as neurodegenerative disease^23^ and alcohol abuse.^24 25^

The consequences of autonomic dysfunction for patients with TBI are potentially profound and far-reaching. Our study found a significant burden of autonomic symptoms, which correlated with other clinical symptoms as well as a negative view of their current health status and pessimistic view of future health. Worse quality of life and psychological distress is reported in otherwise healthy patients with vasovagal syncope or POTS and neurological patients with concurrent autonomic dysfunction.^26–28^ Additionally, CAN regions are also important for cognition and emotional processing,^29 30^ which means that autonomic dysfunction may be an important biomarker for or contributor to post-TBI cognitive/psychological dysfunction. Furthermore, interoception, the ‘signalling and perception of internal bodily sensations’ is important for both cognitive and emotional function.^31 32^ Post-TBI autonomic dysfunction may lead to abnormal bodily responses to cognitive or emotional stimuli,^3^ which could contribute to post-TBI cognitive/psychological morbidity.

### Limitations

The autonomic function testing cohort was retrospectively recruited. This means that we only captured patients whose physicians had already considered autonomic dysfunction to be a possible cause of their symptoms, which were usually orthostatic symptoms. The prevalence of abnormal testing may be lower in an unselected population, however the prospective cohort suggests that there is likely to be a significant proportion. Future large-scale prospective studies could address this.

Patients filled out the COMPASS-31 irrespective of whether they initially reported symptoms, so there is a possible risk of over-reporting by patients when presented with a list of symptoms rather than free recall. However, given that patients’ scores were significantly higher than non-TBI controls, this is unlikely to be a significant factor in our results.

For largely practical reasons, we only included msTBI in our cohorts, so our findings may not be generalisable to mild/repetitive TBI. Autonomic dysfunction has been reported in mild TBI^33^ so future studies should seek to include a mild TBI cohort if possible.

## Conclusion

We present evidence for clinically relevant, subjective and objective, autonomic dysfunction after msTBI. We hope these findings increases awareness of autonomic dysfunction after TBI, and promote more diagnostic engagement. Future work should further characterise the nature of post-TBI autonomic dysfunction, investigate how TBI causes these problems, and the links between autonomic dysfunction and other post-TBI sequelae.

## Data Availability

Data are available on reasonable request. Raw scores from the COMPASS31 questionnaire and anonymised clinical autonomic function testing reports from the retrospective cohort are available on request to the corresponding author.

## References

[1] Benarroch EE. Physiology and pathophysiology of the autonomic nervous system. Continuum 2020;26:12–24. 10.1212/CON.0000000000000817 31996619

[2] Hilz MJ, Wang R, Markus J, et al. Severity of traumatic brain injury correlates with long-term cardiovascular autonomic dysfunction. J Neurol 2017;264:1956–67. 10.1007/s00415-017-8581-1 28770375 PMC5587629

[3] Amorapanth PX, Aluru V, Stone J, et al. Traumatic brain injury results in altered physiologic, but not subjective responses to emotional stimuli. Brain Inj 2018;32:1712–9. 10.1080/02699052.2018.1519598 30261156

[4] Pertab JL, Merkley TL, Cramond AJ, et al. Concussion and the autonomic nervous system: an introduction to the field and the results of a systematic review. NeuroRehabilitation 2018;42:397–427. 10.3233/NRE-172298 29660949 PMC6027940

[5] Purkayastha S, Stokes M, Bell KR. Autonomic nervous system dysfunction in mild traumatic brain injury: a review of related pathophysiology and symptoms. Brain Inj 2019;33:1129–36. 10.1080/02699052.2019.1631488 31216903

[6] Sletten DM, Suarez GA, Low PA, et al. Compass 31: a refined and abbreviated composite autonomic symptom score. Mayo Clin Proc 2012;87:1196–201. 10.1016/j.mayocp.2012.10.013 23218087 PMC3541923

[7] Low PA, Tomalia VA, Park K-J. Autonomic function tests: some clinical applications. J Clin Neurol 2013;9:1. 10.3988/jcn.2013.9.1.1 23346153 PMC3543903

[8] Meyfroidt G, Baguley IJ, Menon DK. Paroxysmal sympathetic hyperactivity: the storm after acute brain injury. Lancet Neurol 2017;16:721–9. 10.1016/S1474-4422(17)30259-4 28816118

[9] Malec JF, Brown AW, Leibson CL, et al. The Mayo classification system for traumatic brain injury severity. J Neurotrauma 2007;24:1417–24. 10.1089/neu.2006.0245 17892404

[10] Autonomic Failure. 1. Oxford university press, 2013.

[11] Cheshire WP, Freeman R, Gibbons CH, et al. Electrodiagnostic assessment of the autonomic nervous system: a consensus statement endorsed by the American autonomic Society, American Academy of Neurology, and the International Federation of clinical neurophysiology. Clinical Neurophysiology 2021;132:666–82. 10.1016/j.clinph.2020.11.024 33419664

[12] Rea NA, Campbell CL, Cortez MM. Quantitative assessment of autonomic symptom burden in postural tachycardia syndrome (POTS). J Neurol Sci 2017;377:35–41. 10.1016/j.jns.2017.03.032 28477704

[13] Dipaola F, Barberi C, Castelnuovo E, et al. Time course of autonomic symptoms in postural orthostatic tachycardia syndrome (POTS) patients: two-year follow-up results. Int J Environ Res Public Health 2020;17. 10.3390/ijerph17165872. [Epub ahead of print: 13 08 2020]. PMC7460485 32823577

[14] Koay S, Vichayanrat E, Bremner F, et al. Multimodal biomarkers quantify recovery in autoimmune autonomic ganglionopathy. Ann Neurol 2021;89:753–68. 10.1002/ana.26018 33438240

[15] D'Amato C, Greco C, Lombardo G, et al. The diagnostic usefulness of the combined COMPASS 31 questionnaire and electrochemical skin conductance for diabetic cardiovascular autonomic neuropathy and diabetic polyneuropathy. J Peripher Nerv Syst 2020;25:44–53. 10.1111/jns.12366 31985124

[16] Kim Y, Seok JM, Park J, et al. The composite autonomic symptom scale 31 is a useful screening tool for patients with parkinsonism. PLoS One 2017;12:e0180744. 10.1371/journal.pone.0180744 28683089 PMC5500372

[17] Ruet A, Bayen E, Jourdan C, et al. A detailed overview of long-term outcomes in severe traumatic brain injury eight years post-injury. Front Neurol 2019;10:120. 10.3389/fneur.2019.00120 30846966 PMC6393327

[18] Kanjwal K, Karabin B, Kanjwal Y, et al. Autonomic dysfunction presenting as postural tachycardia syndrome following traumatic brain injury. Cardiol J 2010;17:482–7. 20865679

[19] Johnson VE, Stewart W, Smith DH. Axonal pathology in traumatic brain injury. Exp Neurol 2013;246:35–43. 10.1016/j.expneurol.2012.01.013 22285252 PMC3979341

[20] Hill CS, Coleman MP, Menon DK. Traumatic axonal injury: mechanisms and translational opportunities. Trends Neurosci 2016;39:311–24. 10.1016/j.tins.2016.03.002 27040729 PMC5405046

[21] Jenkins PO, Mehta MA, Sharp DJ. Catecholamines and cognition after traumatic brain injury. Brain 2016;139:2345–71. 10.1093/brain/aww128 27256296 PMC4995357

[22] De Simoni S, Jenkins PO, Bourke NJ, et al. Altered caudate connectivity is associated with executive dysfunction after traumatic brain injury. Brain 2018;141:148–64. 10.1093/brain/awx309 29186356 PMC5837394

[23] Wilson L, Stewart W, Dams-O'Connor K, et al. The chronic and evolving neurological consequences of traumatic brain injury. Lancet Neurol 2017;16:813–25. 10.1016/S1474-4422(17)30279-X 28920887 PMC9336016

[24] Julian TH, Syeed R, Glascow N, et al. Alcohol-Induced autonomic dysfunction: a systematic review. Clin Auton Res 2020;30:29–41. 10.1007/s10286-019-00618-8 31222483 PMC6987055

[25] Weil ZM, Corrigan JD, Karelina K. Alcohol use disorder and traumatic brain injury. Alcohol Res 2018;39:171–80. 31198656 10.35946/arcr.v39.2.07 PMC6561403

[26] Benrud-Larson LM, Dewar MS, Sandroni P, et al. Quality of life in patients with postural tachycardia syndrome. Mayo Clin Proc 2002;77:531–7. 10.4065/77.6.531 12059122

[27] Ng J, Sheldon RS, Ritchie D, et al. Reduced quality of life and greater psychological distress in vasovagal syncope patients compared to healthy individuals. Pacing Clin Electrophysiol 2019;42:180-188. 10.1111/pace.13559 30488466 PMC6358504

[28] Tomic S, Rajkovaca I, Pekic V. Impact of autonomic dysfunctions on the quality of life in Parkinson's disease patients. Acta Neurol Belg 2017;117:207–11. 10.1007/s13760-016-0739-6 28028676

[29] Critchley HD, Mathias CJ, Josephs O, et al. Human cingulate cortex and autonomic control: converging neuroimaging and clinical evidence. Brain 2003;126:2139–52. 10.1093/brain/awg216 12821513

[30] Neudorfer C, Elias GJB, Jakobs M, et al. Mapping autonomic, mood and cognitive effects of hypothalamic region deep brain stimulation. Brain 2021;144:2837–51. 10.1093/brain/awab170 33905474 PMC8557336

[31] Kreibig SD. Autonomic nervous system activity in emotion: a review. Biol Psychol 2010;84:394–421. 10.1016/j.biopsycho.2010.03.010 20371374

[32] Garfinkel SN, Seth AK, Barrett AB, et al. Knowing your own heart: distinguishing interoceptive accuracy from interoceptive awareness. Biol Psychol 2015;104:65–74. 10.1016/j.biopsycho.2014.11.004 25451381

[33] Esterov D, Greenwald BD. Autonomic dysfunction after mild traumatic brain injury. Brain Sci 2017;7. 10.3390/brainsci7080100. [Epub ahead of print: 11 08 2017]. PMC5575620 28800081

