# Supplementary material for: Autonomic dysfunction after moderate-to-severe traumatic brain injury: symptom spectrum and clinical testing outcomes
Source: BMJ Neurol Open. 2022 Apr 24;4(1):e000308. doi: 10.1136/bmjno-2022-000308 (PMC9039351; doi:10.1136/bmjno-2022-000308)
Supplement: Supplementary data [file bmjno-2022-000308supp001.pdf]

TABLE 1: Clinical characteristics of recruited participants

(A) Prospective Symptom Assessment Cohort – TBI patients					
**ID**	**Sex**	**Age range at assessment**	**Time since injury (months)**	**Mechanism of injury**	**Medications**
P1*	M	30-39	7	Fall/Assault	*nil*
P2*	F	17-19	8	Fall	Vit D
P3	M	20-29	7	RTA	Vit D, Vit B12
P4*	M	20-29	8	Fall	Propranolol, terbinafine, sumatriptan PRN, Vit D
P5	M	20-29	20	RTA	Salbutamol INH, citalopram, codydramol PRN
P6	M	20-29	20	Assault	Vit D
P7	M	40-49	19	RTA	*nil*
P8	F	40-49	13	Fall	Sertraline
P9	M	40-49	27	RTA	Citalopram, Vit D
P10	M	40-49	22	Fall	*Nil*
P11	M	40-49	23	Fall	Clonazepam, esomeprazole, lactulose PRN, paracetamol
P12	M	20-29	12	*nk*	*nil*
P13	M	20-29	37	Sport	Paracetamol
P14*	M	40-49	6	Seizure	Lamotrigine, folate, thiamine, Vit B12, paracetamol, codeine PRN, sertraline
P15*	F	40-49	6	RTA	*nil*
P16	M	20-29	7	Fall	Dihydrocodeine PRN
P17*	M	50-59	6	Object	Ondansetron, paracetamol
P18	F	30-39	21	RTA	Thyroxine
P19*	F	70-79	72	RTA	Sertraline, atorvastatin, melatonin, omperazole, oxybutynin, Adcal D3, trimethoprim, paracetamol, cinnarizine
P20	F	40-49	25	RTA	*nil*
P21*	M	17-19	21	Fall	*nil*
P22*	M	70-79	6	Fall	Apixaban, thyroxine
P23*	M	50-59	103	RTA	Valproate, levetiracetam, clobazam, phenofibrate, rosuvastatin, B12

P24	M	30-39	13	Assault	Vit D
P25	M	70-79	22	Fall	Levetiracetam, Lacosamide, rivaroxaban, aspirin, bisoprolol, atorvastatin, Vit D, Vit B12
P26	M	40-49	105	*nk*	Tramadol, fluxetoine, Fostair, carbocystine, fexofenadine, beclomethasone, montelukast, salbutamol
P27*	M	40-49	81	Fall	Sertraline, melatonin
P28*	M	30-39	10	RTA	Co-dydramol, ocular lubricant, paracetamol, potassium bicarb + potassium chloride
P29*	F	30-39	6	Fall	Amitriptylline
P30	M	30-39	64	Assault	Codeine PRN
P31*	F	50-59	12	RTA	Amitriptylline
P32	F	50-59	26	Fall	Amitriptylline, propranolol, paracetamol, ramipril, amlodipine
P33*	M	30-39	38	Assault	Sertraline
P34*	M	20-29	6	RTA	Over the counter “painkillers” PRN
P35	M	30-39	66	RTC	*Nil*
P36	M	50-59	299	RTC	Lamotrigine, oxybutynine
P37	M	50-59	9	Object hit head	*Nil*
P38	F	70-79	11	RTC	Quinine, aciclovir, pregabalin
P39*	M	17-19	8	RTC	*Nil*

*indicates participants who also answered the Subjective Rating of General Health Status question

**(B) Prospective Symptom Assessment Cohort – non-TBI Controls**			
**ID**	**Sex**	**Age range at assessment**	**Medications**
C1	F	40-49	*Nil*
C2	M	50-59	*Nil*
C3	M	20-29	*Nil*
C4	F	20-29	*Nil*
C5	F	20-29	Sertraline
C6	F	20-29	*Nil*
C7	F	20-29	Combined oral contraceptive pill

C8	M	20-29	Duloxetine
C9	M	20-29	*Nil*
C10	F	20-29	Vitamin D, zinc, fish oil supplements
C11	F	20-29	*Nil*
C12	M	20-29	Vitamin D
C13	M	20-29	*Nil*
C14	F	30-39	Combined oral contraceptive pill
C15	F	30-39	Iron supplements, multivitamins, Vitamin B
C16	F	30-39	*Nil*
C17	F	30-39	*Nil*
C18	F	30-39	*Nil*
C19	F	30-39	Combined oral contraceptive pill, Vitamin D, multivitamins, probiotics
C20	M	30-39	*Nil*
C21	M	30-39	*Nil*
C22	M	30-39	*Nil*
C23	M	40-49	Naproxen, Codeine
C24	M	40-49	*Nil*
C25	F	40-49	*Nil*
C26	F	40-49	*Nil*
C27	M	40-49	*Nil*
C28	F	50-59	*Nil*
C29	F	50-59	*Nil*
C30	M	50-59	*Nil*
C31	F	30-39	*Nil*
C32	M	50-59	*Nil*
C33	F	50-59	*Nil*
C34	F	50-59	*Nil*
C35	M	60-69	*Nil*
C36	F	60-69	*Nil*

C37	F	60-69	*Nil*
C38	M	60-69	*Nil*
C39	M	60-69	*Nil*
C40	M	60-69	Multivitamins, Vitamin D
C41	M	40-49	*Nil*
C42	M	60-69	*Nil*
C43	M	60-69	*Nil*
C44	M	70-79	*Nil*

**(C) Retrospective Autonomic Function Testing Cohort**						
**ID**	**Sex**	**Age range at injury (time since injury in months)**	**Age range at assessment (length of symptoms in months)**	**Mechanism of injury**	**Symptoms leading to referral**	**Medications** (not stopped for testing)
R1	F	30-39 (309)	60-69 (20)	Fall off bike	Dizziness, presyncope, tinnitus, problems with focussing vision, episodic severe photosensitivity	Salbutamol INH
R2	M	20-29 (416)	60-69 (47)	RTA	Unsteady, dizziness, “problems with vision”, falls with LOC, nausea	Carbamazepine, Levetiracetam, Pregabalin, Clopidogrel, Atorvastatin, Clobazam, Vitamin D
R3	M	20-29 (20)	20-29 (17)	RTA	LOC episodes with tunnel vision, no seizure indicators	*Nil*
R4	F	20-29 (44)	30-39 (44)	RTA	Constipation, headaches, poor sleep, dizziness and lightheaded, urinary frequency and urgency, temperature dysregulation and flushes on wakening	Thyroxine, Venlafaxine, OCP, Ferrous Fumarate, Cholecalciferol, Rizatriptan, Laxido, Flunarizine

R5	M	30-39 (159)	40-49 (105), 40-49 (127)	*nk*	Collapse with LOC on standing but not meals (thoroughly investigated for seizures), headaches	Tramadol PRN, Ibuprofen PRN, codydramol PRN, paroxetine
R6	F	30-39 (123)	40-49 (123)	RTA	Collapses, sometimes with headache after	*Nil*
R7	F	50-59 (41)	50-59 (41)	RTA	Anhidrosis, pain and headache, frequent falls with occasional LOC, poor balance, nausea on standing, alternating constipation/ diarrhoea, poor short-term memory	*Nil*
R8	F	10-19 (35)	20-29 (35)	*nk*	Dizziness and lightheaded on standing, palpitations and chest pain, panic attacks - all worsened on standing/exertion, constipation	Omeprazole, gabapentin, paracetamol, mebeverine, Oramorph PRN
R9	M	10-19 (65)	20-29 (65)	RTA	Easy, and painful erections (urological investigations normal)	*Nil*
R10	F	10-19 (314)	40-49 (38)	RTA	Hyperhidrosis, presyncope while standing, dizziness when hot and standing, dry mouth and eyes	Amitripylline, cocodamol, gabapentin, omeprazole, ocybutynin, tramadol, venalfaxin
R11	M	40-49 (46)	50-59 (46)	RTA	Cold hands and feet, increased IBS symptoms, dizziness (persisting though diminished after vestibular manoeuvres)	Laxido
R12	M	20-29 (50)	30-39 (14)	RTA	Always cold, episode of sweating in hands and feet, dizziness when sitting	Quetiapine, clonazepam, fexofenadine, fluticasone, beclomethasone, valproate, Vit D, sildeafil, fluexetine, salbutamol
R13	M	30-39 (137)	40-49 (137)	RTA	Hyperhidrosis, nocturia, excessive thirst, constipation, dizziness and visual disturbance when standing, occasional posture-related palpitations	Codeine, lefopramine, beconase, valproate, symbicort INH, fluticasone INH, salbutamol INH, sodium valproate, ibuprofen

R14	F	40-49 (2)	40-49 (2)	RTA	Two episodes of collapse with LOC (with normal EEG and cardiac investigations)	*Nil*
R15	M	30-39 (36)	39-39 (36)	*nk*	Fatigue, anosmia/ ageusia, LOC with rapid recovery but possible seizure activity noted on two episodes, memory/ cognition deficits	*Nil*
R16	M	40-49 (33)	40-49 (33)	Assault	Constantly feeling cold, R leg has temperature sensory deficit	*Nil*
R17	M	30-39 (150)	40-49 (150)	Fall	Lightheaded on standing	Paracetamol PRN
R18	M	40-49 (194)	50-59 (194)	RTA	Erectile dysfunction, nocturia and some incontinence, postural lightheadedness, profuse exertional upper body sweating, falls with LOC and syncope markers	Carbamazepine, Clonidine (started for sweating), Calcichew D3

KEY : *nk* = not known, RTA = road traffic accident, EEG = electroencephalogram, LOC = loss of consciousness, INH = inhaler, PRN = as required medication. Note that age decades are given for anonymisation purposes.

[1] carried out in conjunction with autonomic tests due to symptoms
